# Development and Evaluation of a Novel Anti-Ageing Cream Based on Hyaluronic Acid and Other Innovative Cosmetic Actives

**DOI:** 10.3390/polym15204134

**Published:** 2023-10-18

**Authors:** Anca Maria Juncan, Claudiu Morgovan, Luca-Liviu Rus, Felicia Loghin

**Affiliations:** 1 Department of Toxicology, Faculty of Pharmacy, “Iuliu Hațieganu” University of Medicine and Pharmacy, 6 Pasteur Str., 400349 Cluj-Napoca, Romania; floghin@umfcluj.ro; 2 SC Aviva Cosmetics SRL, 71A Kövari Str., 400217 Cluj-Napoca, Romania; 3 Preclinic Department, Faculty of Medicine, “Lucian Blaga” University of Sibiu, 2A Lucian Blaga Str., 550169 Sibiu, Romania; liviu.rus@ulbsibiu.ro

**Keywords:** hyaluronic acid, anti-ageing cream, active cosmetic ingredients, safety assessment, in silico evaluation, efficacy studies

## Abstract

The importance of incorporating hyaluronic acid (HA) as a cosmetic ingredient in skin care formulations emerged lately because the amount of HA naturally found in the epidermis decreases with age, and when applied to the skin through cosmetic products, it confers hydration and reduces the appearance of wrinkles. Currently, the diversity of cosmetic products for mature skin and the use of various and innovative active ingredients supporting their anti-ageing effect represent ample proof that the cosmetic industry is currently relying on these actives. The main objective of this study was the development of an anti-ageing formulation, incorporating HA and different other active ingredients. The developed formulation contains a novel complex of natural waxes, with an essential role in the restoration of the skin’s hydro–lipid barrier, in combination with innovative active ingredients—like low-molecular hyaluronic acid (LMW-HA), sodium hyaluronate (NaHA), ectoin, gold, and an anti-ageing botanical complex—contributing to optimal skin hydration specifically designed to reduce the visible signs of ageing. An important objective was represented by the skin compatibility and topography assessment after 28 days (D28) of regular application of the developed cream. Stability testing, physicochemical characteristics, and microbiological control, including efficacy testing of the used preservative (challenge test) were performed for the cosmetic formulation. In silico approaches were applied to demonstrate the safety of cosmetic-related substances and the risk assessment of the cosmetic formulation. Safety and instrumental evaluation were performed to demonstrate the skin tolerance—the compatibility and the efficacy, respectively—of the developed anti-ageing cream. As result, quality control of the developed cosmetic formulation evidenced an appropriate cosmetic preparation with desirable aspect and adequate physicochemical characteristics. The concentrations of restricted ingredients like preservatives and UV filters were in accordance with those recommended by the Regulation (EC) No. 1223/2009 and so were considered to be safe. Additionally, according to the margin of safety (MoS) calculation, cosmetic ingredients incorporated in the developed formulation could be considered safe. The developed formulation was very well tolerated, and wrinkle depth and length in the periorbital area were significantly reduced after 28-day cosmetic treatment. Subjects’ assessment questionnaires revealed self-perceived benefits referring to the cosmetic qualities and efficacy of the anti-ageing cream. This study confirmed the skin tolerance and efficacy of the new complex anti-ageing cream incorporating HA, microencapsulated sodium hyaluronate, ectoin, and a botanical extract. The formulated cosmetic product could serve as a daily care for mature skin to alleviate the effects of skin ageing.

## 1. Introduction

Skin ageing is a multifaceted phenomenon that takes place gradually over a period of several decades. In addition to endogenous factors, different environmental influences sustain the skin ageing process. Skin functionality declines, resulting in the appearance of various conditions and diverse skin diseases, which can seriously diminish quality of life. Acknowledging the pathophysiology of skin ageing and taking precautionary measures to prevent skin damage represents the initial step towards achieving a healthy ageing process [1]. 

**Figure 1:**
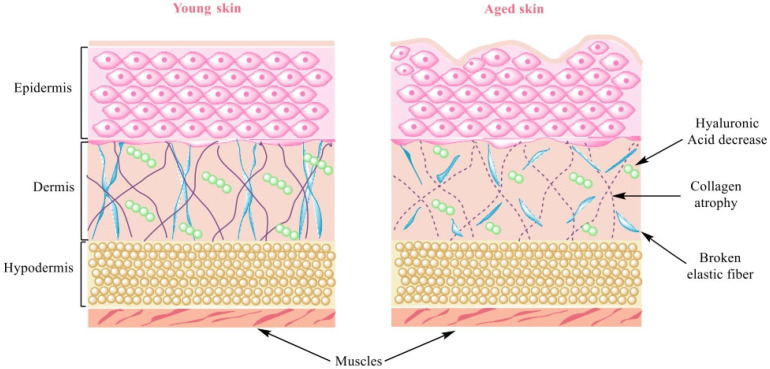
Comparison between young and aged skin. Wrinkle formation due to loss of collagen, elastin, and GAGs (glycosaminoglycans, e.g., HA).

Optimal health includes improving the appearance of aged skin, with the possibility to use not only safer and more effective active ingredients but also cosmetic products and procedures to diminish non-aesthetically appealing skin appearance. Wrinkles occur in the areas in which the dermal proteins collagen and elastin are less dense and the bond between the dermis and epidermis is weaker [2]. Types I and III are fibril-forming collagens. The most abundant collagens in the skin are fibril types I (70–75%) and III (18–21%) and types IV and VII, which are situated in the basement membrane and form anchoring fibrils [3]. Fibroblasts are very important for the skin, as they are essential for the production of hyaluronic acid (HA); collagen types I, III, and VII (which are essential for the skin); and elastin. The activity of fibroblasts is substantial for the elasticity and firmness of the skin, and it can be concluded that in aged skin, type I and III collagen decrease. Additionally, hyaluronic acid content and elastin functions are significantly reduced [4] (Figure 1). 

Atrophy of dermal collagen (especially the reticular dermis) is observed in intrinsically aged skin, while photoaged skin exhibits a significant degradation of fibrillar collagen and a specific distribution of type VII collagen from the dermoepidermal junction (DEJ) [5].

Further research in cosmetology will lead to a higher proficiency for risk exposure assessment but also to supplying and providing an optimal nutritional basis for healthy skin ageing and to the development of products that prevent or even inverse negative epigenetic modifications [6]. The use of innovative active ingredients and the diversity of cosmetic products that support an anti-ageing effect is ample proof that the cosmetic industry is currently relying on this ingredient category. A multitude of cosmetics that are commercially available possess moisturizing effect or aim to improve the signs of skin ageing [7].

The value of the global market for anti-ageing products was USD 20.25 billion in 2018. The annual growth rate until is estimated to be 5.4% [8]. The increasing share of the global elderly population, along with technological advances in the cosmetic industry, are key factors driving the growth of the anti-ageing products market. Anti-wrinkle products are gaining importance through innovative formulations to slow down the ageing process. The most preferred active ingredients are retinoids, HA, and alpha hydroxy acids, while serums and creams are considered the most attractive product type/formulation by consumers at the moment [9]. 

HA and its derivates are widely used in cosmetic formulations, considering that the interest in using HA as an active ingredient in cosmetic products occurred with the discovery that the level of hyaluronan naturally present in the dermis diminishes with age, and incorporating it into skin care formulations can help to maintain skin moisture, minimize fine wrinkles, and enhance skin texture and elasticity. HA possesses various effects that renders it superior to other cosmetic ingredients, providing skin regeneration, moisturizing, and anti-ageing properties [10,11]. The biological activity and skin penetration of HA are dependent on the molecular weight of this active and show various beneficial effects on the skin [12,13]. 

Currently, commercially available products with hydrolysed HA represent a reduced percentage compared to cosmetics containing its salts (such as sodium hyaluronate NaHA) in their formulation. In the coming period, the greatest increase in the HA market is rated to be in Europe and Asia.

Commercially available cosmetics contain HA or its derivates in association with other actives, e.g., peptides, probiotics, proteins, amino acids, vitamins, plant extracts, etc. These complementary actives enhance the quality and efficacy of the cosmetic formulation, providing supplementary benefits [13]. 

The main objective of the present study was to develop a novel anti-ageing cosmetic formulation, incorporating an innovative complex of natural waxes together with active ingredients like low molecular HA (LMW-HA); microencapsulated NaHA, ectoin, gold (microspheres); and an anti-ageing plant extract with complementary anti-ageing effect. An important part of the study was represented by quality control by determining physicochemical characteristics and microbiological testing, including the preservative efficacy evaluation (challenge test) of the developed formulation. Skin compatibility evaluation together with the assessment of the cosmetic’s tolerance at the site of application (repeated application) and efficacy assessment of the cosmetic formulation were also performed.

## 2. Materials and Methods

### 2.1. Cosmetic Ingredients and Actives Selection for the Development of an Anti-Ageing Formulation

In accordance with the material safety data sheets (MSDS), a prioritization and classification of the studied cosmetic ingredients was carried out to provide a solid basis for the selection of raw materials for the product base that was formulated—emulsifiers, emollients, viscosity modifiers, pH modifiers, preservatives, etc. The formulation was generically denominated “Anti-ageing cream”.

The complex of three functionalized waxes based on jojoba, mimosa, and sunflower wax (INCI (International Nomenclature of Cosmetic Ingredients) *Jojoba* esters and *Acacia Decurrens* flower wax and *Helianthus Annuus* (sunflower) seed wax and polyglycerin-3 (Acticire^®^ MB, Gattefosse, Saint Priest, France) acts as an emollient, bringing softness to the skin [14].

Octyldodecyl myristate (MOD MB, Gattefosse, France) and caprylic/capric triglyceride (Labrafac CC, Gattefosse, France) are liquid emollients for oils and non-comedogenic emulsions which provide a rich feel and improve the spreadability on the skin [15,16]. 

Cetyl alcohol, glyceryl stearate, PEG-75 stearate, ceteth-20, steareth-2 (Emulium^®^ Delta MB, Gattefosse, France) are O/W (oil/water) emulsifiers with excellent sensory characteristics [17]. 

Hydrogenated ethylhexyl olivate combined with hydrogenated olive oil unsaponifiables (Softolive, Givaudan, Paris, France) is an olive-based emollient representing a natural alternative to silicone. It restores skin’s softness while imparting a unique sensory profile to cosmetic formulations [18].

Phenoxyethanol (and) Ethylhexylglycerin (Euxyl PE 9010, Schülke&Mayr GmbH, Norderstedt, Germany) is a cosmetic preservative in liquid form with a broad spectrum against bacteria, yeasts, and mould fungi [19]. 

Carbomer (Carbopol^®^ ETD 2050, Lubrizol Advanced Materials, Inc., Cleveland, OH, USA) is an easily dispersible white powder polymer. This cross-linked polyacrylic acid homopolymer is polymerized with a cosolvent system and is preferred due to its toxicological and safety profile. It is highly effective as a thickening and suspending ingredient at low concentrations [20]. 

Sodium polyacrylate, hydrogenated polydecene, and Trideceth-6 (RapiThix A-60, Ashland, OR, USA) are rheology modifiers based on sodium polyacrylate and are designed for ease of use and efficient thickening properties, resulting in elegant lotions and creams with silky sheer-thinning properties [21].

Butyl methoxydibenzoylmethane (Eusolex^®^ 9020, Merck, Darmstadt, Germany) and octocrylene (Eusolex^®^ OCR, Merck, Germany) are excellent UV-A and UV-B filters, respectively. They are safe for use and have versatile applications [22,23].

For the selection of the active ingredients, the documentation and related efficacy studies were reviewed. Low-molecular-weight HA (LMW-HA) (PrimalHyal 50, Givaudan, France) and microencapsulated NaHA (Hyalusphere PF, Givaudan, France) (Table 1) [24,25,26], 22 k gold microcapsules (Unispehres Gold, Givaudan, Kemptthal, Switzerland) [27], ectoin (RonaCare^®^ Ectoin, Merck, Germany) [28,29], and a pigment (RonaFlair^®^ Flawless, Merck, Germany) with mattifying and anti-ageing effects [30] were selected as active ingredients. Additionally, a botanical active derived from horse chestnut flowers—fructose, glycerine, water, and *Aesculus Hippocastanum* (horse chestnut) extract—was incorporated into the cosmetic formulation. It possesses anti-ageing effects, especially smoothing the eye contour area and acting on periorbital wrinkles. The botanical extract was obtained via extraction of horse chestnut (*Aesculus hippocastanum*) flowers using an innovative solvent composed of fructose, glycerine, and water [31,32]. According to the technical data sheet (TDS) of the botanical active, it is specified as an amber to red limpid liquid with medium-intensity honeyed note, pH 4.0 to 6.0, refractive index (at 20 °C) 1.445 to 1.475, and a glycosylated flavols content ≥ 500 mg/kg.

### 2.2. Development and Formulation of the Anti-Ageing Cream—Preparation Procedure

Phase A: The oil phase contains the following cosmetic ingredients, which were melted at 75–80 °C, in descending order of melting points: *Jojoba* esters, *Helianthus Annuus* (sunflower) seed wax, *Acacia Decurrens* flower wax, polyglycerin-3, octyldodecyl myristate, caprylic/capric triglyceride, hydrogenated ethylhexyl olivate, hydrogenated olive oil unsaponifiables, cetyl alcohol, glyceryl stearate, PEG-75 stearate, ceteth-20, and steareth-20, butyl methoxydibenzoylmethane, and octocrylene.

Phase B: The aqueous phase incorporating ultrapure water (INCI Aqua, PURELAB^®^ Option Q7 (Type I), ELGA LabWater, High Wycombe, UK), glycerine (Elton Corporation S.A., Ilfov, Romania), and the preservative was heated to 75 °C. The carbomer and triethanolamine (Stera Chemicals SRL, Jilava, Romania) were added, and homogenization for complete dispersion of the ingredients was performed. Phase A was added under continuous stirring over Phase B using an T 50 digital ULTRA-TURRAX with a S 50 N-G 45 G dispersing tool (IKA, Staufen, Germany).

Phase C contains the viscosity modifier ingredients sodium polyacrylate, hydrogenated polydecene, trideceth-6, previously homogenized with 10 mL of the total water content. A part of the active ingredients fructose, glycerine, water, *Aesculus Hippocastanum* (horse chestnut) extract, ectoin, and the pigment were added to the emulsion obtained from phase A and B cooled to 40 °C. After that, the hydrolysed HA, previously dissolved in 5 mL of water for complete dissolution, was added to the emulsion. NaHA microcapsules together with the 22 k gold microcapsules and the fragrance (Luminating Skin Care Ecoboost HICC MOD, CPL Aromas, Bishop’s Stortford, UK) were added to the emulsion and homogeneously dispersed in the cream mass, avoiding microcapsule breakage. 

### 2.3. Evaluation of the PhysicoChemical and Microbiological Characteristics of the Developed Formulation

In accordance with the current legislation, namely Regulation (EC) No. 1223/2009 of the European Parliament and of the Council of 30 November 2009 on cosmetic products a series of tests was carried out: (I) stability testing of the cosmetic formulation [33,34,35]; (II) physicochemical control, organoleptic testing (appearance, colour, odour), and determination of the pH, density and viscosity of the developed formulation [36,37]; (III) microbiological evaluation for the assessment of microbiological contamination and preservative efficacy test (challenge test) [38,39,40,41,42]. 

#### 2.3.1. Stability Testing of the Cosmetic Formulation

The accelerated stability study was performed for a period of 30 days under conditions of product storage at 4 °C (LKUv 1610 MediLine, Liebherr, Germany), 20 °C, and 40 °C (Natural convection drying oven SLN-32 (STD), Pol-Eko, Wodzisław Śląski, Poland).

#### 2.3.2. Quality Control of the Cosmetic Formulation

The quality control of the developed formulation consisted of the following determinations: physicochemical control; organoleptic testing (appearance, colour, odour) (ISO 6658:2005 p. 5.4.2 [43]); determination of pH (PB-234 ed. I of 03.10.2013r.), density (20 °C) (PB-155 ed.I of 2 May 2012), and viscosity (Brookfield DV-III Ultra, spindle Sc4-25/RPM: 0.5 (o/min/shear rate 0.11 (1/s))) of the developed formulation.

#### 2.3.3. Microbiological Control and Assessment of the Effectiveness of the Preservation of the Cosmetic Formulation

Antimicrobial protection was evaluated for the developed formulation using standard procedures: enumeration and detection of aerobic mesophilic bacteria (SR EN ISO 21149:2017 [44]), yeast and mould count (SR EN ISO 16212:2017 [45]), Staphylococcus aureus detection (SR EN ISO 22718:2016 [46]), Candida albicans detection (SR EN ISO 18416:2016 [47]), Escherichia coli detection (SR EN ISO 21150:2016 [48]), and Pseudomonas aeruginosa detection (SR EN ISO 22717:2016 [49]). Assessment of the effectiveness and quality of the preservation system (phenoxyethanol and ethylhexylglycerin) of the cosmetic formulation was also performed using the international cosmetics challenge test standard (PN EN ISO 11930:2012 [50]).

### 2.4. In Silico Approaches for Safety Evaluation of Cosmetic-Related Substances and Risk Assessment of the Cosmetic Formulation

A new software for integrated hazard and exposure assessment for risk assessment of cosmetic ingredients (SpheraCosmolife v. 0.24) [51] was used for in silico evaluation of the formulation’s cosmetic ingredients. This software provides searches through internal database considering if the substance is listed in any of the Annexes of the Cosmetic Regulation (EC) No. 1223/2009. It also calculates external dermal exposure and internal exposure (systemic exposure dose) via modelling Kp (skin permeability coefficient), providing hazard identification for different toxicological endpoints. The system searches experimental values for NOAEL (no observed adverse effect level), provides model predicted values, calculating the margin of safety (MoS) and the threshold of toxicological concern (TTC) using a Cramer decision tree [52]. 

The MoS, which represents the ratio of the NOAEL to the systemic exposure dose (SED), must be higher than or equal to 100 (10 × 10^−10^ signifies the interspecies extrapolation (from animal to human) and 10 for intraspecies differences (age, gender, body weight, ethnicity)). Both values can be subdivided into toxicokinetic and toxicodynamic factors [53]. A cosmetic ingredient is considered safe if the MoS is higher or equal to 100.

### 2.5. Safety Evaluation (Skin Tolerance) and Efficacy Assessment of the Cosmetic Formulation

#### 2.5.1. Dermatological Semi-Open Test

The aim of the study was the assessment of sensitizing/irritant potential and the skin tolerance of the developed formulation on healthy human skin under a patch test, evaluating the erythema and skin oedema level after product application.

A panel of 25 healthy subjects—Caucasians, with phototype I–IV according to the Fitzpatrick scale, without any prior allergic reactions and any skin irritations or conditions that require medication—participated in the study. None of the volunteers previously reported any hypersensitivity or adverse reactions to the formulation’s individual ingredients.

The product was applied under a patch (12 mm diameter Finn Chamber, SmartPractice, USA) on the arm or interscapular area. Two control samples (a “blind” control sample and a control sample with water) were applied to avoid any inaccurate interpretations related to skin irritations. The patch was removed 48 h after the application, and a dermatologist examined the skin response 30 min after patch removal; 72 h after the application, the skin was examined for a response, and in case of any irritations that could arise or persisted for 72 h following the application, a supplementary evaluation was carried out after 96 h. The result is expressed based on the Average Irritation Index (X_av_), and considering this, the product was classified as “not-irritating”, “slightly irritating”, “moderately irritating”, or “highly irritating” [54,55,56].

#### 2.5.2. Use Test and Instrumental Test under Dermatological Control

##### In Use test with dermatological control

The evaluation focused on the assessment of the cosmetic formulation’s tolerance at the application site based on regular, repetitive application of the product (repetitive test 28 days). In total, 25 subjects—women, all skin types—were included in the subject panel for skin tolerance evaluation. Subjects participating in the study applied the product twice daily under normal conditions of use of the cosmetic formulation and were evaluated by a dermatologist initially and at the end of the study. The site of product application was without irritation and changes requiring pharmacological treatment [57,58,59,60].

##### Instrumental Test for Wrinkle Length and Depth

The aim of this evaluation was the assessment of the cosmetic formulation effect on the reduction of wrinkle length and depth (Visioline^®^ VL 650, Courage + Khazaka, Köln, Germany). The study included 10 subjects—women, aged 30–70 years, all skin types. The evaluation has been performed at the site of application before (D0) and after 28 days (D28) of regular use of the cosmetic formulation [61,62,63,64,65]. 

##### In Vivo Determination of the Sun Protection Factor (SPF)

The sun protection factor (SPF) assessment represents a method for evaluating the protection provided by cosmetic products containing UV filters against erythema (reddening of the skin) caused by solar ultraviolet rays on human skin, utilizing a simulator with a xenon arc lamp of specific and defined output (Solar Light type Single Port 16S-150-001, Solar Light Co. Erythema DCS detector PMA2105, Solar Light Co., Glenside, PA, USA). The SPF evaluation was assessed on 10 healthy subjects—Caucasians with phototypes I–III according to the Fitzpatrick scale, untanned on the test area (the back is the chosen anatomical region for the test area). 

A skin area of each subject is exposed to UV without any protection, and another area is exposed after application of a test sun protection product. A third area is exposed after application of a SPF reference sunscreen formulation. By increasing the UV dose, varying degrees of skin erythema (redness due to superficial vasodilation) are generated. These delayed erythemal responses are visually assessed for redness intensity 16 to 24 h after UV radiation. The minimum erythemal dose (MED) for unprotected skin (MEDu) and the MED obtained after the application of a sun protection product (i.e., the MED for product protected skin, MEDp) must be determined on the same subject on the same day. An individual sun protection factor (SPFi) for each subject tested is calculated as the ratio of MEDp/MEDu. The SPF for the tested formulation is the arithmetic mean of all valid SPFi results from each subject in the test and is expressed to one decimal place [66,67,68,69,70]. 

##### Assessment of the Effect Claimed for the Cosmetic Product

A self-assessment study was carried out based on questionnaires regarding subjects’ appreciation of the cosmetic quality and efficacy of the tested formulation. All 25 subjects included in the study received a self-assessment questionnaire comprising 12-questions associated with the product’s characteristics (e.g., products compliance and satisfaction) and efficacy using a 5-grade evaluation scale (definitely no, no, neutral, yes, definitely yes). 

All subjects included in the safety and efficacy evaluation signed an informed consent form (ICF), which included information about the study’s purpose, methodology, and potential side effects. The studies were conducted by an external laboratory (J.S. Hamilton Poland Sp. z o.o., Gdynia, Poland) according to the most recent recommendations of the Regulation of the European Parliament and of the Council (EC) No. 1223/2009 of 30 November 2009 on Cosmetic Products, Cosmetics Europe, The Personal Care Association (previously COLIPA), Guidelines Product Test Guidelines for the Assessment of Human Skin Compatibility (1997), Guidelines for the Evaluation of the Efficacy of Cosmetic Products (2008), and International Sun Protection Factor Test Method (2006).

## 3. Results

### 3.1. Development and Formulation of the Anti-Ageing Cream

Emollient, emulsifier, viscosity modifier, and preservative type ingredients were incorporated and were selected for their compatibility as well as for the stability of the anti-ageing cream. MSDS of the categories of cosmetic ingredients containing data on the chemical name, physicochemical properties, toxicological studies, compatibilities with other cosmetic ingredients, concentration limits, etc., were reviewed, and the basis for the cosmetic formulations was established. Active ingredients selection was based on the claim of the finished product—LMW-HA and NaHA in encapsulated form were used, together with other active ingredients, and can claim complementary beneficial effects—a botanical complex, ectoine, and 22 k gold microcapsules.

Table 2 includes the commercial name of the ingredients, the INCI denomination, the role of the ingredient in the cosmetic formulation, the supplier of each ingredient and the concentration limit (INCI-KEY).

### 3.2. Quality Control of the Anti-Ageing Cream—Stability Testing, Physicochemical and Microbiological Assessment

An important study is the quality control of the developed formulation by determining the appropriate physicochemical and pharmacotechnical characteristics (pH, viscosity). The obtained results showed that the studied formulation is stable and revealed a preparation with an acceptable cosmetic appearance and adequate physicochemical and pharmacotechnical characteristics. The results of the physicochemical determinations of the developed cosmetic formulation are presented in Table 3.

**Figure 2:**
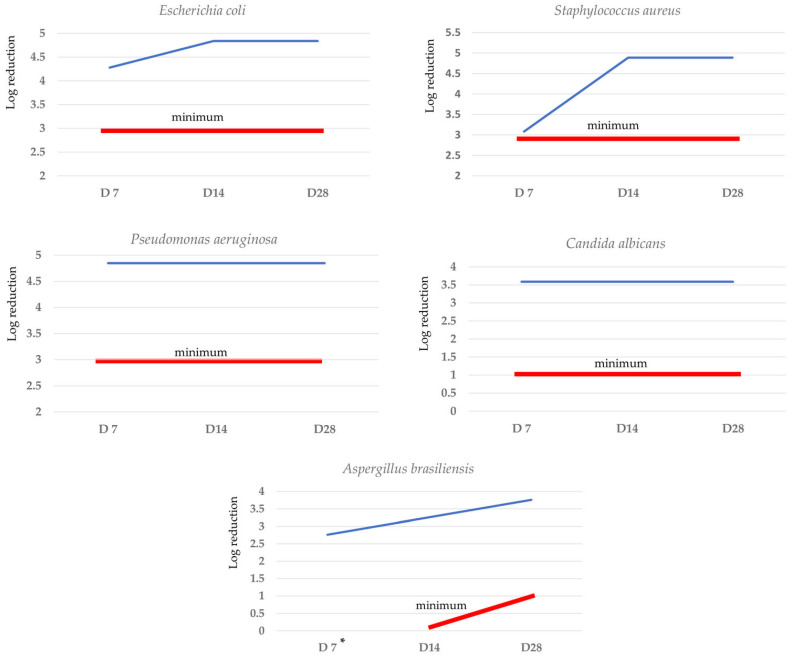
Challenge test results for the developed anti-ageing cream. * No criteria; D7—after 7 days, D14—after 14 days; D28—after 28 days. **−−−** effectiveness of the antimicrobial protection, **−−−** minimum effectiveness recommended by standards.

The results of the microbiological determinations of the developed formulation are reported in Table 4, confirming the microbiological quality of the anti-ageing Cream:

The performed challenge test has confirmed the effectiveness of the antimicrobial protection of the cosmetic formulation. The results of the challenge test are shown in Figure 2. 

### 3.3. In Silico Approaches for the Safety Evaluation of Cosmetic-Related Substances and Risk Assessment of the Cosmetic Formulation

According to Regulation (EC) No. 1223/2009, safety assessment of cosmetics relies on the safety of the ingredients. Thus, the chemical structure, toxicological data, and exposure must be known. The safety assessment for cosmetic ingredients is performed based on the concept of the risk assessment process generally implemented for chemicals, which comprises hazard identification, exposure, and dose-response assessment. Based on the exposure assessment of a chemical used as a cosmetic ingredient, human exposure is evaluated. For the assessment, the declared functions of the ingredient, its application, the concentration in different cosmetic product categories and the frequency of use are considered. All this information is available from the SCCS Notes of Guidance for the Testing of Cosmetic Ingredients and Their Safety Evaluation, (NoG, 11th Revision SCCS/1628/21, adopted 30–31 March 2021). 

Table 5 presents the summary of the results for all the cosmetic ingredients in the developed formulation. The results are corelated with the product category and the ingredient concentration ranges introduced into the system. In Table 5, hazard and exposure characteristics of the anti-ageing cream ingredients are presented. Data on the following information are included: (i) the presence of an ingredient in an Annex of the Cosmetics Regulation, (ii) mutagenicity (Ames test), (iii) skin senzitisation, (iv) the dermal absorption (according to the Kroes approach), (v) the MoS, and (vi). the TTC. 

The used database identified information related to the exposure of each ingredient based on the product category and ingredient concentration. The calculation of exposure is carried out for various situations. The parameters specified by the SCCS NoG for the product category are of greatest importance for the determination of the external exposure. The SED (in mg/kg bw/d, where the human body weight (bw) is considered as a default value of 60 kg) is determined for three cases: (1) absorption of 100%, oral or inhalation exposure; (2) absorption of 50%, a default value for dermal exposure, as stated also by the SCCS NoG; or (3) the more realistic option for dermal absorption based on the models for skin permeation. 

To demonstrate the safety of the ingredients of the developed formulation, a risk characterization was performed given the systemic toxicity and the MoS. The software also featured an evaluation utilizing the TTC approach, in which the external and internal exposure data are compared with the TTC threshold for the specific ingredient considering the dermal bioavailability of cosmetics.

### 3.4. Safety Evaluation (Skin Tolerance) and Efficacy Assessment of the Cosmetic Formulation

#### 3.4.1. Dermatological Semi-Open Test

The performed study allows us to conclude that the developed anti-ageing cream used by the subjects did not cause any hypersensitivity. Additionally, no previous adverse reactions to cosmetic ingredients were reported. In the test panel of subjects, irritations or allergic reactions were not observed, leading to a conclusion that the product meets the criteria of skin compatibility. The average irritation index (Xav) was determined as the sum of negative reactions (erythema and oedema) and the product was classified as “not irritating” according to X_av_ < 0.50 (Table 6). This demonstrated that the cosmetic formulation was well tolerated by the skin.

#### 3.4.2. Use Test and Instrumental Test under Dermatological Control

##### In Use Test with Dermatological Control

Based on examinations and interviews collected from subjects it was found that the tested formulation was very well tolerated at the application site. In all subjects who finished the study (subject No. 2 finished the study at day 24 instead of day 28, and subject No. 6 did not finish the study) during the regular application, there were no negative symptoms that evidenced any intolerance to any ingredient of the product, such as irritation, burning sensation, redness, or itching.

##### Instrumental Test Result for Wrinkle Length and Depth

The efficacy testing aimed to demonstrate the claimed effect of the developed formulation. The skin microrelief was analysed by evaluating the periorbital wrinkles before and after cosmetic treatment, and determination of the SPF was performed for the anti-ageing cream.

**Figure 3:**
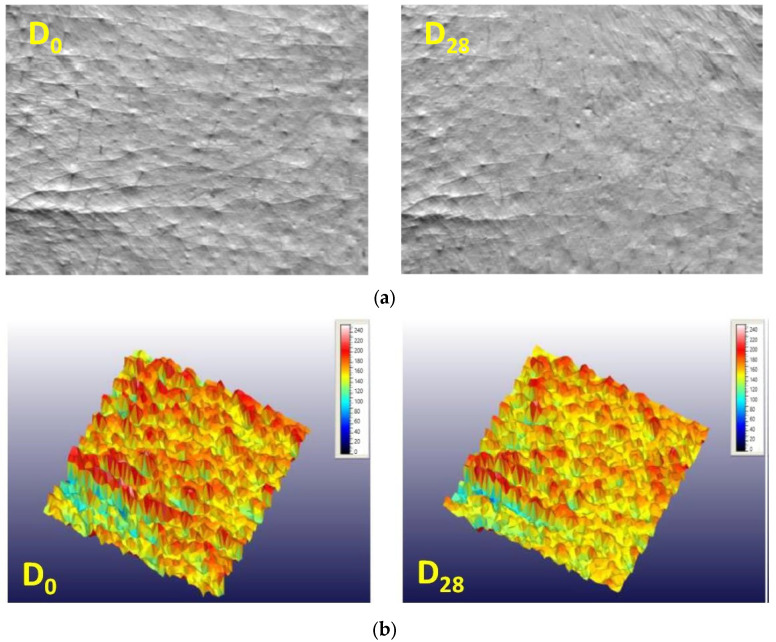
Skin surface evaluation (skin microrelief) before (D0) and after 28 days (D28) of the anti-ageing cream. Images are presented for Subject No. 10: (**a**). skin replicas at D0 and D28 and (**b**) 3D pictures at D0 and D28).

**Figure 4:**
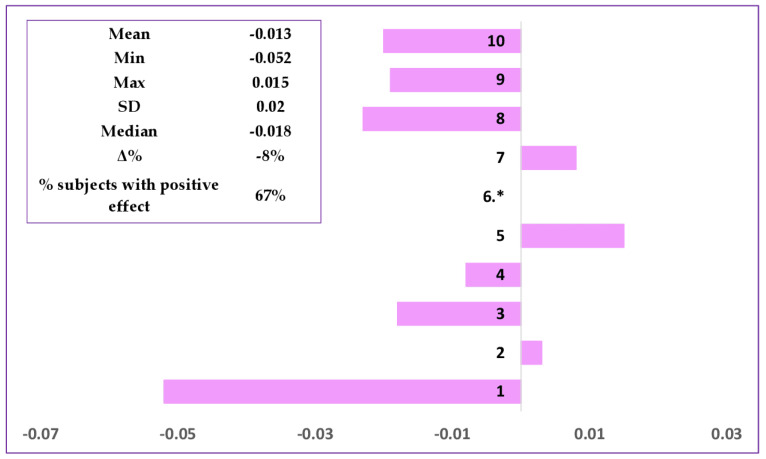
Evaluation of wrinkles length (in mm) at D0 (before product application) (D0) and at D28 (after 28 days of regular application). * The result was not included in the calculation for Subject No. 6 (untraceable results).

**Figure 5:**
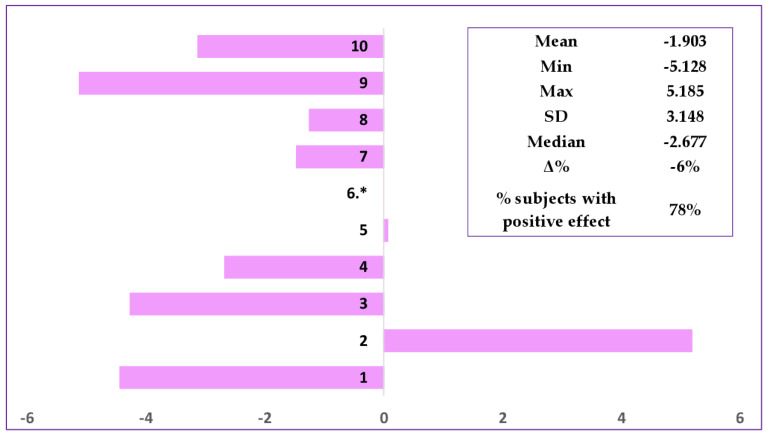
Evaluation of wrinkles depth (in μm) at D0 (before product application) (D0) and at D28 (after 28 days of regular application. * The result was not included in the calculation for Subject No. 6 (untraceable results).

The developed formulation was evaluated before and after 28 days of repeated applications on the face area, and the cosmetic effect was assessed under normal conditions of use. The skin microrelief was evaluated by image analysis demonstrating significant improvement of wrinkles. Figure 3 shows the skin microrelief images for the subject with one of the most representative results (Subject No. 10), illustrating skin improvement after application of the cosmetic formulation. Comparison of the images (skin replicas ((a) before cosmetic treatment and after cosmetic treatment) and 3D imaging ((b) before cosmetic treatment and after cosmetic treatment) of the results determined for the skin treated with the anti-ageing cream evidenced noticeable changes to the skin microrelief. Through skin microrelief analysis, it was demonstrated that the product reduces wrinkle length (67% subjects with positive response, Δ = −8%) and depth (78% subjects with positive response, Δ = −6%) when the parameters value decrease (Figure 4 and Figure 5).

##### In Vivo Evaluation of the SPF Factor

SPF determination showed that the average SPF value is 16.0, so the SPF value that can be claimed on the cosmetic formulation (according to 2006/647/EC) is 15 (average protection) (Table 7).

##### Assessment of the Claimed Effect for the Cosmetic Formulation

**Figure 6:**
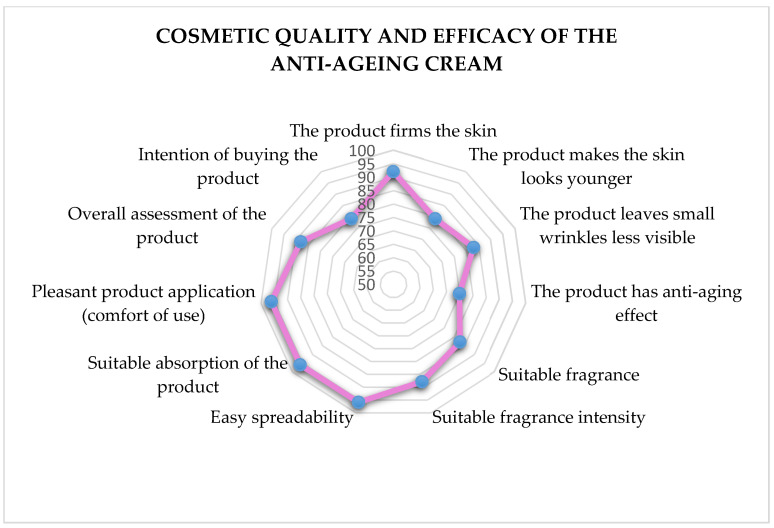
Self-assessment questionnaire regarding the anti-ageing cream’s cosmetic quality and efficacy (data are expressed as percentage of positive answers at the end of the cosmetic treatment period (D28).

According to the self-assessment study, based on a questionnaire on the subjects’ assessment of the product properties, all subjects evaluated the tested formulation with a significant percentage of positive answers related to the product’s compliance and efficacy (Figure 6).

## 4. Discussion

The World Health Organization (WHO) defines “active aging” as the process of maximizing possibilities for health, participation, and security to improve the life quality as individuals age [71]. Some authors discuss the relevance of the universality of human ageing, considering that ageing is not a disease but rather a “special form” of condition [72]. Others suggest that ageing is a gradual decrease in tissue function that could lead to mortality [73,74].

Apart from chronological ageing, the extrinsic factors, such as changing lifestyle, UV exposure, environmental pollution, lack of sleep, stress, and genetics, are main factors which cause skin ageing and affect the firmness, elasticity, structural support, and skin integrity [75].

The safety of cosmetics is crucial since they are frequently applied to sensitive body parts, without medical supervision, and/or for extended time periods. Safety includes the product’s composition, packaging, and information. The efficacy of cosmetics refers to the capacity of providing the claimed effect. The desired properties of a cosmetic formulation can be achieved by using appropriate active ingredients claiming the efficacy of the product together with the adequate excipients [76]. Regulation (EC) No. 1223/2009 states the importance of the safety assessment of cosmetics and of the general aspects which ensure their quality, efficacy, and safety.

In this study, innovative emollient ingredients were selected for the development of an anti-ageing cosmetic formulation. Some traditional emollients currently used in cosmetics and currently commercially available on the market (e.g., Stearic Alcohol, Cetyl Alcohol, Petrolatum, and/or mineral oil) were replaced with a natural complex of waxes and natural oils [77,78,79,80]. This innovative emollient complex has perfect skin compatibility and confers the claimed effect of the anti-ageing formulation. The selection of active ingredients was also based on the claim of the formulation. Thus, LMW-HA and encapsulated NaHA were incorporated, together with other active ingredients, which can claim complementary beneficial effects—a botanical complex, ectoin, and 22 k gold microcapsules.

**Figure 7:**
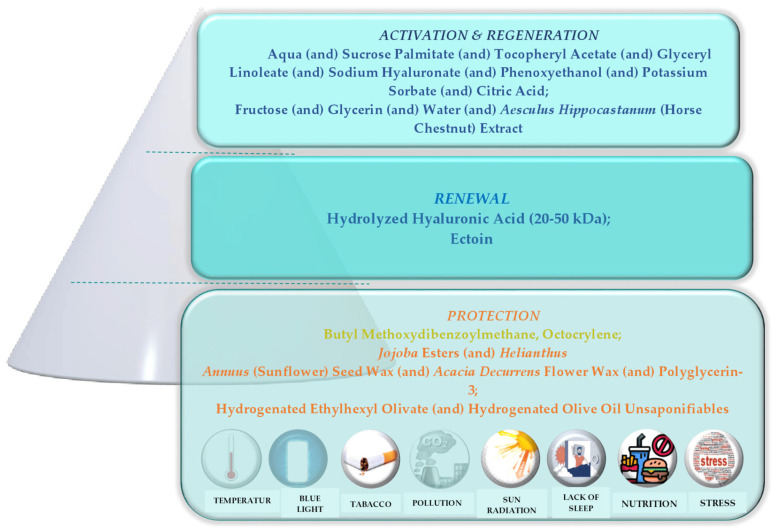
Skin health and beauty pyramid with environmental factors with evidence of active ingredients incorporated in the developed anti-ageing cream according to their cosmetic claim—protection at the base of the pyramid, renewal in the middle, and activation/regeneration on the top of the pyramid.

According to the literature data, the skin pyramid is a valuable concept for cosmeceutical skin care ingredients. For superior skin renewal from the outside to the inner skin layers, all three levels of the pyramid must be considered. The pyramid’s base denotes protection, including regeneration and sun protection; the centre evidences rejuvenation, moisturization, and cell turnover; and the top includes activation and skin regeneration [81]. The skin health and beauty pyramid with environmental and lifestyle aggressors, emphasizing the active ingredients incorporated in the developed anti-ageing cream, is shown in Figure 7.

An important step in the development of a cosmetic formulation is stability evaluation, considering significant parameters to assure the safety of the product with the purpose of predicting organoleptic, physicochemical, and microbiological modifications that are fundamental to the initial development of cosmetics [82,83]. The developed formulation showed stability for the organoleptic characteristics, pH, and viscosity values.

The preservative phenoxyethanol and ethylhexylglycerin were used to preserve the developed cosmetic formulation (0.5–1.0% in emulsion-type cosmetic products) [84,85,86]. The preservative is indicated for use in cosmetics due to the fact that it is efficient in low concentrations, it is pH independent, and it offers a broad spectrum of action.

In the EU, microbiological assessment requires the use of *Pseudomonas aeruginosa*, *Staphylococcus aureus,* and *Candida albicans* from official collection strains and additionally should include specific germs which are known to lead to spoilage of cosmetic products. The Regulation on cosmetic products (EC No. 1223/2009) itself does not specify the test procedure for the challenge test. In-house test protocols have been long established in addition to tests from the EU and US Pharmacopoeia [41,87]. 

According to the results of the microbial and challenge studies, the efficacy of the preservative and its concentration in the formulation and the antimicrobial protection of the developed cosmetic product were confirmed in the study.

According to Regulation EC No. 1223/2009, data collection on the safety of cosmetic ingredients is mandatory for the risk assessment of cosmetics. Considering the intended product use conditions, cosmetic products and all incorporated ingredients should be safe. The cosmetics legislation includes restrictions on the testing and marketing of finished cosmetics and their ingredients, especially in respect to toxicity tests using animals. As a result, it became of crucial importance to have available animal-free methodologies for safety evaluation. Safety information for new and innovative cosmetic ingredients can be obtained from validated (or scientifically valid) alternative methods. Beginning on 11 March 2013, animal-free methodology, also known as New Approach Methodology (NAM), is the only method permitted for the testing of cosmetics. According to these features, the SCCS considers the following aspects for the safety assessment: Annex substances of Regulation (EC) 1223/2009 (hair dyes/colorants (Annex III/IV), preservatives (Annex V), UV filters (Annex VI) all available toxicological data—including in chemico data (physicochemical properties) and in silico (computational) models for quantitative structure activity relationship (QSAR) and read-across, respectively—and physiologically based pharmacokinetics (PBPK) and toxicokinetics (PBTK) modelling. The appropriate application of in silico models can provide a useful non-animal alternative for the estimation of toxicity of cosmetic ingredients and products, with the possibility to predict safe concentration limits of cosmetic ingredients [53,88,89,90,91,92].

For example, the SpheraCosmolife system used for the safety evaluation of cosmetic ingredients of the anti-ageing cream in this study is based on several equations and in silico models, for estimation of exposure or to predict hazards. The system may evaluate the risk related to the formulations ingredients by merging the results of various models, considering the availability of experimental values from the system database, or providing predictions.

The internal exposure for the corresponding uptake routes (dermal, oral, inhalation) can be derived from the external exposure. Based on the external exposure, it is possible to calculate the internal or systemic exposure. For the determination of the SED, the absorption specific to the respective exposure route must be considered. In the safety assessment process, the MoS of the cosmetic ingredient is based on this internal dose, namely the SED. In the case of the cosmetic ingredients selected to develop the anti-ageing cream, the calculated MoS was higher than 100. Normally, when the MoS of an ingredient is ≥100, it can be considered to be safe. 

For example, a particular case that should be discussed is the preservative phenoxyethanol, which is listed in Annex V of Regulation (EC) 1223/2009 and may be used at a maximum concentration of 1%. The concentration of phenoxyethanol in the developed formulation is 0.9%, which is lower than 1% and is safe and in compliance with the law. Additionally, the MoS of phenoxyethanol calculated by the software is ≥100, so it can be considered to be safe.

The cases of butylmethoxydibenzoylmethane (BMBM) (1-(4-tert-butylphenyl)-3-(4-methoxyphenyl) propane-1,3-dione) and avobenzone are other interesting examples of safety and risk assessment. BMBM is frequently used as a UVA filter in cosmetics and personal care cosmetics (with a maximum concentration of 5%—Annex VI of the Regulation (EC) 1223/2009). According to the literature data, avobenzone has been evaluated extensively in toxicologic studies and has a favourable safety profile. As part of the European Union’s Registration, Evaluation, Authorization, and Restriction of Chemicals (REACH) regulation, a current and extensive safety dossier exists for avobenzone on the European Chemicals Agency (ECHA) website that can be used to support its incorporation in cosmetics at a level of 5%. Its safety has been reviewed and found to be acceptable for use in sunscreens at levels of 5% by the authoritative regulatory bodies such as the SCCS [69,93,94,95,96]. The stabilization of avobenzone could be achieved by using the correct stabilizer, e.g., another filter like octocrylene, which is also incorporated in the developed formulation with the aim of achieving a SPF of 15 [69]. 

Based on a preliminary in silico safety assessment with a risk characterization and an MoS determination of BMBM using a dermal absorption rate of 40%, it was concluded that the application of avobenzone as a UV filter at a concentration level of up to 2% in the developed formulation could cause a risk to consumers (MoS = 51.11). To demonstrate the safety of the UVA filter, the calculation, and assumptions to ascertain a MoS≥ 100 for 2% avobenzone used in the developed formulation is presented Table 8. The MoS calculation for BMBM was performed, taking into consideration a NOAEL of 450 mg/kg bw/day, based on a sub-chronic repeated oral dose toxicity study [96,97] in rats with a determined SED of 0.2414 mg/kg bw/day. In this case, the calculated MoS >> 100 demonstrated that avobenzone was acceptable for use at a concentration of 2% in the developed formulation.

In addition, the clinical safety evaluations performed for the developed formulation allow us to conclude that the anti-ageing cream respects the requirements of the skin compatibility assessment, so it can be classified as “not irritating” and is very well tolerated at the application site.

In this study, the efficacy assessment results confirmed the anti-ageing effect of the evaluated formulation by reducing wrinkles length and wrinkles depth in the periorbital area. Under the study conditions, based on instrumental evaluation and subject’s self-assessment, it was confirmed that the product firms the skin, provides a younger skin aspect, leaves small wrinkles less visible, and has a general anti-ageing effect. According to the SPF evaluation for the developed formulation, a mean SPF value of 16.0 was confirmed, which states a SPF value of 15 (medium protection).

## 5. Conclusions

Optimal health includes improving the appearance of aged skin, with the main objective of diminishing non-aesthetically appealing aspects, and it is now to use safer and more effective cosmetic ingredients and products.

In this study, the results confirmed, according to stability testing and physicochemical characteristics, an acceptable cosmetic preparation with optimal appearance and appropriate physicochemical characteristics. Microbiological control, including efficacy testing of the used preservative (challenge test) were also confirmed for the developed formulation. 

Risk assessment was performed for the safety evaluation of cosmetic ingredients with the aid of in silico approaches. Concentrations of restricted ingredients—phenoxyethanol as a preservative and butylmethoxydibenzoylmethane as a UVA filter—incorporated in the formulation were in accordance with those recommended by the Regulation (EC) No. 1223/2009 and so were considered and confirmed to be safe.

The combination of HA together with different actives in this novel anti-ageing cream improved skin texture after 28 days of cosmetic treatment. In conclusion, clinical evaluation of an anti-ageing cream containing LMW-HA, encapsulated NaHA, ectoin, 22 k microcapsules, and a botanical extract showed global improvements in periorbital skin condition, significant wrinkle length, and depth reduction. This test formulation was highly rated by subjects for efficacy and product attributes and was very well tolerated. This complex cream could give further assistance to the continuous treatment of mature skin.

Further research could sustain the risk assessment and provide an optimal basis for healthy skin ageing and cosmetics that would be able to block or reverse negative epigenetic changes.

## Figures and Tables

**Table 1:** Cosmetic claim depending on HA MW/HA derivates used in the anti-ageing formulation.

Commercial Name	INCI Name	Molecular Weight	Cosmetic Claim
Primalhyal 50	Hydrolysed Hyaluronic Acid	20–50 kDa	firming, anti-ageing
Hyalusphere PF	Aqua, Sucrose Palmitate, Tocopheryl Acetate, Glyceryl Linoleate, Sodium Hyaluronate, Phenoxyethanol, Potassium Sorbate, and Citric Acid	/	anti-ageing

**Table 2:** Formulation of the developed Anti-Ageing Cream.

Commercial Name	INCI	Function	Supplier	INCI-KEY * (%)
Acticire MB	*Jojoba* Esters, *Helianthus* *Annuus* (Sunflower) Seed Wax (and) *Acacia Decurrens* Flower Wax, and Polyglycerin-3	emollient	Gattefossé	E
MOD MB	Octyldodecyl Myristate	emollient	Gattefossé	E
Labrafac CC	Caprylic/Capric Triglyceride	emollient	Gattefossé	E
Softolive	Hydrogenated Ethylhexyl Olivate (and) Hydrogenated Olive Oil Unsaponifiables	emollient	Givaudan Active Beauty	D
Emulium Delta MB	Cetyl Alcohol (and) Glyceryl Stearate (and) PEG-75 Stearate (and) Ceteth-20 (and) Steareth-2	emulsifier	Gattefossé	E
Eusolex 9020	Butyl Methoxydibenzoyl methane	UVA filter	Merck	E
Eusolex OCR	Octocrylene	UVB filter	Merck	D
Aqua	Water	solvent		A
Glycerol	Glycerin	denaturant/humectant/solvent	ELTON	E
Carbopol ETD 2050	Carbomer	emulsion stabilising/viscosity controlling/gel forming	Lubrizol	F
Euxyl PE 9010	Phenoxyethanol and Ethylhexylglycerin	preservative	Schülke & Mayr GmbH	F
TEA	Triethanolamine	buffering agent	ELTON	G
Rapithix A-60	Sodium Polyacrylate, Hydrogenated Polydecene, and Trideceth-6	viscosity controlling/binding/film forming	Barentz	F
PrimalHyal™ 50	Hydrolysed Hyaluronic Acid	antistatic/humectant/skin conditioning/moisturising	Givaudan Active Beauty	F
Hyalusphere PF	Aqua, Sucrose Palmitate, Tocopheryl Acetate, Glyceryl Linoleate, Sodium Hyaluronate, Phenoxyethanol, Potassium Sorbate, and Citric Acid	active ingredient/anti-wrinkle	Givaudan Active Beauty	E
Luxury Unispheres Gold	Mannitol Cellulose Calcium Sodium Borosilicate CI 77492 (US: Iron Oxides) Silica CI 77891 (US: Titanium Dioxide) CI 77480 (Gold) Tin Oxide Hydroxypropyl Methylcellulose	active ingredient/shimmer effect	Givaudan Active Beauty	E
Gatuline Link n Lift	Fructose (and) Glycerin (and) Water (and) *Aesculus Hippocastanum* (Horse Chestnut) Extract	active ingredient/anti-ageing	Gattefossé	E
Rona Flair Flawless	Silica (and) CI 77891 (and) CI 77491	functional filler/anti-wrinkle/anti-ageing agent	Merck	E
Luminating Skin Care Eco-Boost HICC MOD	Perfume	deodorant/masking	CPL	F

* INCI Key A > 50%; 5% < D ≤ 10%; 1% < E ≤ 5%; 0.1% < F < 1%; G < 0.1%.

**Table 3:** Physicochemical characteristics of the Anti-Ageing Cream.

Test	Unit	Result
Viscosity at 20 °C (Brookfield DV-III Ultra)	mPa·s	412.2 × 10^3^ ± 9 × 10^3^
Density at 20 °C (PB-155 ed.I of 2 May 2012)	g/cm^3^	1.002 ± 0.003
Organoleptic testing (ISO 6658:2005 p. 5.4.2)		
Appearance		Homogeneous emulsion *
Color		Light beige (with gold microcapsules)
Odor		Specific
Consistency		Specific of emulsion
pH (PB-234 ed. I of 03.10.2013r.)		5.9 ± 0.2

* Nonmechanical impurities.

**Table 4:** Microbiological assay of the Anti-Ageing Cream.

Parameter	ISO Standard	Result (CFU/g)	Permissible Limits (CFU/g)	Concordance
Enumeration and detection of aerobic mesophilic bacteria	21149:2017	<10	<100	√
Yeast and mould count	16212:2017	<10	<10	√
Staphylococcus aureus detection	22718:2016	0	0	√
Candida albicans detection	18416:2016	0	0	√
Escherichia coli detection	21150:2016	0	0	√
Pseudomonas aeruginosa detection	22717:2016	0	0	√

CFU—colony forming units.

**Table 5:** Hazard and exposure specifications of the cosmetic ingredients incorporated in the developed formulation. Product type: anti-ageing cream. Ingredients.

Ingredient Id	CAS	INCI	Conc. %	Annex	Mutagenicity	Skin Sensitization	Dermal Abs.	MoS	TTC
*Jojoba* Esters	68953	*Jojoba* Esters	3.75	-	-	-	-	-	
*Helianthus Annuus* (Sunflower) Seed Wax	8001-21-6	*Helianthus Annuus* (Sunflower) Seed Wax	0.5	-	-	-	-	-	
*Acacia Decurrens* Flower Wax	98903-76-5	*Acacia Decurrens* Flower Wax	0.05	-	-	-	-	-	
Polyglycerin-3	25618-55-7	Polyglycerin-3	0.05	-	NM (e.v.)	NS (e.v.)	80%	26,725.35	0.046 mg/kg bw/day
Octyldodecyl Myristate	22766-83-2	Octyldodecyl Myristate	1.0	-	NM (+++)	S (+)	80%	1345.12	0.046 mg/kg bw/day
Caprylic/Capric Triglyceride	65381-09-1	Caprylic/Capric Triglyceride	3.0	-	-	-	-	-	
Hydrogenated Ethylhexyl Olivate	22047-49-0	Hydrogenated Ethylhexyl Olivate	5.0	-	-	-	-	-	
Hydrogenated Olive Oil Unsaponifiables	111-01-3	Hydrogenated Olive Oil Unsaponifiables	2.5	-	-	-	-	-	
Cetyl Alcohol	36653-82-4	Cetyl Alcohol	0.5	-	NM (+++)	S (++)	40%	20,712.51	0.046 mg/kg bw/day
Glyceryl Stearate	31566-31-1	Glyceryl Stearate	0.5	-	NM (+++)	S (+++)	40%	8983.43	0.046 mg/kg bw/day
PEG-75 Stearate	9004-99-3	PEG-75 Stearate	0.25	-	NM (+++)	S (+++)	40%	10,347.97	0.046 mg/kg bw/day
Ceteth-20/Steareth-20	68439-49-6	Ceteth-20/Steareth-20	0.25	-	-	-	-	-	
Butyl Methoxydibenzoylmethane	70356-09-1	Butyl Methoxydibenzoylmethane	2.0	VI	NM (+++)	S (+)	40%	51.11	0.0023 mg/kg bw/day
Octocrylene	6197-30-4	Octocrylene	10.0	VI	NM (e.v.)	S (+)	10%	675.23	0.0023 mg/kg bw/day
Deionized Water	7732-18-5	Aqua	59.2	-	-	-	-	-	
Glycerine	56-81-5	Glycerine	3.0	-	NM (e.v.)	NS (e.v.)	80%	445.42	0.046 mg/kg bw/day
Triethanolamine	102-71-6	Triethanolamine	0.45	III	NM (e.v.)	S (+)	80%	5753.48	0.046 mg/kg bw/day
Carbomer	9007-20-9	Carbomer	0.35	-	NM (e.v.)	S (++)	80%	1597.82	0.0023 mg/kg bw/day
Phenoxyethanol	122-99-6	Phenoxyethanol	0.9	V	NM (e.v.)	NS (e.v.)	80%	460.28	0.046 mg/kg bw/day
Ethylhexylglycerin	70445-33-9	Ethylhexylglycerin	0.1	-	NM (+++)	NS (+)	80%	9737.47	0.0023 mg/kg bw/day
Sodium Polyacrylate	9003-04-7	Sodium Polyacrylate	0.65	-	-	-	-	-	
Hydrogenated Polydecene	68037-01-4	Hydrogenated Polydecene	0.45	-	NM (e.v.)	NS (+)	40%	682.82	0.046 mg/kg bw/day
Trideceth-6	78330-21-9	Trideceth-6	0.06	-	NM (+++)	S (++)	40%	24,235.36	0.046 mg/kg bw/day
Hydrolysed Hyaluronic Acid	9004-61-9	Hydrolysed Hyaluronic Acid	0.5	-	NM (++)	S (+)	10%	13,598,771.33	0.0023 mg/kg bw/day
Sucrose Palmitate	26446-38-8	Sucrose Palmitate	0.25	-	NM (+++)	S (+)	10%	724,425.85	0.0023 mg/kg bw/day
Tocopheryl Acetate	7695-91-2	Tocopheryl Acetate	0.09	-	NM (+++)	S (++)	40%	9153.78	0.0023 mg/kg bw/day
Glyceryl Linoleate	2277-28-3	Glyceryl Linoleate	0.09	-	NM (+++)	S (+++)	40%	49,907.94	0.046 mg/kg bw/day
Sodium Hyaluronate	9067-32-7	Sodium Hyaluronate	0.009	-	-	-	-	-	
Potassium Sorbate	24634-61-5	Potassium Sorbate	0.005	V	NM (e.v.)	S (+++)	40%	1,035,625.52	0.046 mg/kg bw/day
Citric Acid	77-92-9	Citric Acid	0.009	-	NM (e.v.)	NS (++)	80%	752,796.19	0.046 mg/kg bw/day
Fructose	57-48-7	Fructose	2.5	-	NM (+++)	NS (++)	80%	2120.28	0.046 mg/kg bw/day
*Aesculus Hippocastanum* (Horse Chestnut) Extract	8053-39-2	*Aesculus Hippocastanum* (Horse Chestnut) Extract	0.5	-	-	-	-	-	
Silica	7631-86-9	Silica	0.9	-	NM (++)	S (+)	40%	3365.21	0.0023 mg/kg bw/day
CI 77891	13463-67-7	CI 77891	0.24	VI	NM (e.v.)	S (+)	10%	4263.33	0.0023 mg/kg bw/day
CI 77491	1309-37-1	CI 77491	0.02	IV	-	-	-	-	
Ectoin	96702-03-3	Ectoin	0.3	-	M (++)	S (+)	40%	3167.98	0.0023 mg/kg bw/day
Mannitol	69-65-8	Mannitol	0.75	-	NM (e.v.)	S (+)	40%	18,652.44	0.046 mg/kg bw/day
Cellulose	9004-34-6	Cellulose	0.3	-	-	-	-	-	
Calcium Sodium Borosilicate	65997-17-3	Calcium Sodium Borosilicate	0.25	-	-	-	-	-	
CI 77492	51274-00-1	CI 77492	0.05	IV	-	-	-	-	
CI 77891	13463-67-7	CI 77891	0.05	VI	NM (e.v.)	S (+)	10%	20,463.96	0.0023 mg/kg bw/day
Gold	7440-57-5	Gold	0.01	IV	NM (++)	-	10%	107,207.95	0.0023 mg/kg bw/day
Tin Oxide	18282-10-5	Tin Oxide	0.01	-	NM (++)	S (+)	40%	14,415.91	0.0023 mg/kg bw/day
Hydroxypropyl Methylcellulose	9004-65-3	Hydroxypropyl Methylcellulose	0.001	-	NM (++)	S (+)	80%	2,614,954.43	0.046 mg/kg bw/day
Parfum		Parfum	0.1	-	-	-	-	-	

Non-mutagen—NM; mutagen—M; non-sensitizer—NS; sensitizer—S; low reliability—(+); moderate reliability—(++); good reliability—(+++); experimental value—(e.v.).

Non-mutagen: experimental value
Non-mutagen: good (+++) / moderate reliability (++)
Mutagen: moderate reliabity (++)
MoS > 100
MoS < 100










Non-sensitizer: experimental value
Non-sensitizer: low reliability (+)
Sensitizer: good relibility (+++)
Sensitizer: moderate reliability (++)
Sensitizer: low reliability (++)

**Table 6:** Average irritation index (X_av_) expressed as skin response of subjects for the evaluation of irritating and sensitizing effects of the tested formulation.

	T_1_ (48 h after Cosmetic Formulation Application)	T_2_ (72 h after Cosmetic Formulation Application)
**Erythema**	0	0
**Oedema**	0	0
X_av_ *	0	0

* Sum of negative reaction.

**Table 7:** SPF determination for the anti-ageing cream.

Subject’s Characteristics *	MED_u_ (mJ/cm^2^)	MED_s_ (mJ/cm^2^)	SPF_s_ (MED_s_/MED_u_)	MEDp (mJ/cm^2^)	SPF_i p_ (MED_p_/MED_u_)
**S1 (38, M, I)**	21	380	18.1	336	16.0
**S2 (47, M, III)**	25	425	17.0	375	15.0
**S3 (64, W, III)**	26	468	18.0	436.8	16.8
**S4 (30, W, II)**	19	342	18.0	340.5	17.9
**S5 (54, W, II)**	18	306	17.0	302.4	16.8
**S6 (58, W, I)**	16	288	18.0	256	16.0
**S7 (48, W, I)**	15	270	18.0	225	15.0
**S8 (51, W, I)**	16	288	18.0	268.8	16.8
**S9 (42, W, II)**	20	360	18.0	300	15.0
**S10 (56, W, III)**	27	432	16.0	405	15.0
**Average value ± standard deviation**			**17.6 ± 0.7**		**16.0 ± 1.0**

* Age, sex and phototype of the subject’s; I—Fitzpatrick skin phototype I (white skin, always burns, never tans); II—Fitzpatrick skin phototype II (white skin, always burns, minimal tan) and III—Fitzpatrick skin phototype III (white skin, burns minimally, tans moderately and gradually); M—men; W—woman. MED—minimal erythema dose; MEDu—MED for unprotected skin; MEDs—MED for standard product-protected skin; MEDp—MED for product-protected skin; SPFs—individual SPF for standard product-protected skin; SPFi—individual SPF for product-protected.

**Table 8:** Margin of safety calculation for butylmethoxydibenzoylmethane.

Parameter	Value
Amount of product applied daily (SCCS/1628/21 Table 3A)	24.14 mg/kg bw/day
Ingredient Concentration in finished product	2%
Typical body weight of human (bw)	60 kg
Absorption of active ingredient (DAp) (dermal absorption not known, considered as 50%)	50%
Systemic exposure dose (SED)	0.2414 mg/kg bw/day
NOAEL (considering sub chronic oral repeated dose toxicity study, rats)	450 mg/kg bw/day
MoS	NOAEL/SED = 932

## Data Availability

Data are contained within the article.
